# Adverse events following measles-mumps-rubella-varicella vaccine: an independent perspective on Italian pharmacovigilance data

**DOI:** 10.12688/f1000research.26523.2

**Published:** 2021-01-06

**Authors:** Paolo Bellavite, Alberto Donzelli

**Affiliations:** 1 Department of Medicine, University of Verona School of Medicine, Verona, Italy; 2 Fondazione Allineare Sanità e Salute, Milan, Italy

**Keywords:** Measles-mumps-rubella-varicella vaccine, MMRV, Pharmacovigilance, Active surveillance, WHO Guidelines for causality assessment, Vaccine safety, Adverse events following immunisation, AEFI.

## Abstract

Vaccine surveillance programs are crucial for the analysis of the vaccine’s safety profile and the guidance of health policies. The Epidemiological Observatory of the Italian Apulia Region carried out an active surveillance program of adverse effects following immunization (AEFI) after the first dose of the measles-mumps-rubella-varicella (MMRV) vaccine, finding 462 AEFIs per 1000 doses, with 11% rated serious. Applying the World Health Organization (WHO) causality assessment algorithm, 38 serious AEFIs/1000 enrolled were classified as ‘consistent causal associations’ with MMRV immunization. Severe hyperpyrexia, neurological symptoms and gastrointestinal diseases occurred in 38, 20 and 15 cases/1000 enrolled, respectively. A projection of such AEFIs in an Italian birth cohort would give tens of thousands of serious AEFIs. These incidence data are much greater than the incidence of serious AEFIs reported by the Italian Medicines Agency (AIFA) for years 2017 and 2018, mainly based on passive (or mixed) pharmacovigilance. In a previous epidemiological study in the same Italian Region, during an eight year passive surveillance, the reporting rate of serious AEFI was 0.06/1000 doses, and no cases of febrile seizures were detected applying the WHO algorithm. Taken together, the data suggest that passive pharmacovigilance is utterly inadequate to document the real incidence of serious AEFIs and that current methods of assessing causality may be questioned. Active surveillance programs are required in representative population samples, with results presented separately from those of spontaneous reporting, and causality assessment should be performed carefully and using a correct technique for AEFIs presenting as complex and multifactorial diseases, like those with serious neurologic disorders.

## Introduction

Adverse events following immunization (AEFIs) are normally detected by various methods, starting from the preclinical and clinical studies required for product registration and then extending to passive (spontaneous) or active post-marketing surveillance on samples of the population. In fact, the different methods often give different results and a clear view of how many are such events, particularly the most serious ones, is still quite uncertain, especially for the more recently introduced vaccinations. Moreover, a causality assessment is conducted on the adverse events recorded, mostly using the algorithm proposed by World Health Organization (WHO), to discriminate whether an event is related to vaccination or independent. This procedure normally leads to the exclusion of vaccine responsibility in several cases of adverse reactions, attributing the cause to other factors. Recently such procedure, justified to exclude false attributions in the presence of alternative causes, has been criticized because of its uncertainty of application in the more complex and multifactorial diseases ^1, 2^.

*Vaccines* has published a paper by researchers from the University of Bari and the Apulia Region ^3^ reporting and updating the main results of the 2018 report of the Apulia Region official Epidemiological Observatory on surveillance after the administration of measles-mumps-rubella-varicella (MMRV) vaccine ^4^. Subsequently, *Human Vaccines and Immunotherapeutics* reported a retrospective study of AEFIs following MMRV vaccine during eight years of passive pharmacovigilance ^5^. This vaccination has been made mandatory in Italy for all newborns from 2017 onwards. Since 2018, the MMRV vaccine was adopted by six states in Europe, two states in America, and by Australia ^6^.

Despite the results showing a high incidence of serious AEFIs, the authors concluded reassuringly on the safety profile of the MMRV vaccine ^5^ and stated that “ *the active surveillance program confirmed and reinforced the safety profile of the vaccine*" ^3^. A specific aspect concerns the incidence of febrile seizures after vaccination with MMRV. According to the research in Apulia would be 0.5 cases per 1000 follow-ups, much lower than what was reported so far by randomized studies and meta-analyses ^7– 11^. However, this calculation was obtained after discarding 3 out of 4 cases of febrile seizures by applying the WHO causality assessment algorithm. Furthermore, the data of this paper ^3^ are in partial conflict with a retrospective epidemiological research carried out in the same Region with passive methods of AEFI reporting ^5^.

Our article challenges some conclusions of the cited studies ^3– 5^, by analyzing the data and comparing these to those reported by the Italian pharmacovigilance system and by the international literature on the MMRV vaccine. A search was made in PubMed using the key words "MMRV and adverse" (83 papers) or "MMRV and safety" (71 papers) and in the recent Cochrane review on the same topic ^12^ and its bibliography. The data of the incidence of AEFIs reported by the Italian pharmacovigilance system were taken from the reports for years 2017 and 2018, by the Italian Medicines Agency (AIFA), a public body operating under the direction of the Ministry of Health and supervised by the Ministry of Economy. Every year AIFA publishes a report on the vaccine surveillance activities of the previous year. In the reports concerning the vaccinations made in 2017 ^13 ^and in 2018 ^14^, the data of the MMR and MMRV vaccine are aggregated, even if most are related to the MMRV vaccine. Very few cases of AEFIs are reported with the monovalent varicella vaccine. The surveillance system adopted in Italy, and in most other countries, is mainly of the "passive" type, but the data of some "active" studies are also entered.

## Incidence of adverse events

The Apulia active vigilance study ^3 ^enrolled children in the second year of life, for whom there is an active and free vaccination offer of the first dose of MMRV vaccine, after acceptation by the parents, who received diaries for recording any AEFI occurring in the three post-vaccination weeks. The parents were interviewed by telephone 25 days after vaccination, asking them to detail the AEFIs noted in the diaries; data about hospitalizations were also collected. If the AEFIs had not yet resolved, a further follow-up was scheduled one month later. During the years 2017 and 2018, 2,540 children were enrolled, and post-vaccination follow-up was completed for 2,149 subjects (84.6%) ^3^. Among them, 992 AEFIs were detected over the three weeks of monitoring, with a reporting rate of 462/1000 enrolled children.

Among the AEFIs reported, 883 (89.0%) were not serious, while 109 (11.0%) were rated as serious according to the WHO Guidelines ^15^. Events are classified as serious when they result in death, are life-threatening, require in-patient hospitalization or prolongation of existing hospitalization, cause persistent or significant disability/incapacity, are a congenital anomaly/birth defect, or require intervention to prevent permanent impairment or damage. Moreover, in 2016, the AIFA published a list of specific health conditions which, if they occur after a vaccination, must be considered as serious AEFIs (for example, hypotonia-hyporesponsiveness, vasculitis and thrombocytopenia) ^16^.

For serious AEFIs, the authors applied the WHO causality assessment algorithm ^15^, as suggested by AIFA ^16^, to classify AEFIs as "consistent causal association", "inconsistent causal association", "indeterminate", "not-classifiable". The causality assessment was carried out separately by two public health physicians with expertise in vaccinology, and a third physician was consulted in case of disagreement. A causal association consistent with MMRV immunization was classified in 82/109 serious AEFIs (most frequently fever/hyperpyrexia, followed by neurological symptoms and gastrointestinal diseases), with a rate of 38/1000. The authors report that most of the 82 serious events had resolved within 25 days of vaccination, while 10 children (12.2% of the serious AEFIs) had longer-lasting consequences, which had resolved within the following month.

Table 1 displays the main numbers of the Apulia research relating to the serious AEFIs classified as consistent with vaccination. The fourth column shows an extrapolation to an annual birth cohort of 430,000 children of the Italian population. The calculation is indicative and approximate, because it is not certain that the Apulia research sample is representative of an Italian birth cohort. Furthermore, as the authors point out, a sample of just over 2000 subjects cannot reliably detect any rare (>1/10,000 but <1/1000) or very rare (<1/10,000) AEFI. The right column of Table 1 shows the incidence of some serious AEFIs in Italy during 2017, according to the AIFA report ^13^.

**Table 1.** Serious adverse events following immunization (AEFIs) consistently causally associated with measles-mumps-rubella-varicella (MMRV) vaccination according to Stefanizzi *et al*. ^3^ and to the AIFA report for year 2017 ^13^.

	Serious AEFIs from Apulia 2017-2018		Serious AEFIs extrapolated to a cohort of 430,000 children/year	Reporting rate (x 1000 doses) in AIFA report for year 2017
Serious AEFIs	Number (in 2149 children)	Reporting rate (x 1000 enrolled)		
Fever, hyperpyrexia	82	38	16,340	0.108
Neurological symptoms	44	20	8,600	0.002 ^**^
Agitation, nervousness	38	17	7,310	n.r.
Seizures, clonus ^*^	1	0,5	215	0.005
Gastrointestinal diseases	33	15	6,450	n.r.
(Serious) redness, skin rash, swelling, local pain	28	13	5,590	0.02
Lymphadenitis	16	7	3,010	n.r.
Excessive, inconsolable crying	3	1	430	n.r.
Other serious local signs/ symptoms	25	12	5,160	0.007 ^***^
Serious AEFIs persistent more than 25 days	10	4,65	2,000	n.r.

* clonus/febrile seizures were detected in 4/109 serious AEFIs, but only 1 was considered as associated to MMRV vaccination: 2 cases were "not consistently” associated, because of the presence of a not described “alternative cause” of adverse events; 1 case was "indeterminate", because time from vaccination was compatible, but another cause - viral pharyngotonsillitis - was supposed during hospitalization ^3^. N.r.=not reported by AIFA. ** Ataxia/balance disorder. *** Thrombocytopenia.

AIFA, in its 2018 Vaccine Report ^14^, described 0.127 serious events out of 1000 doses of MMRV or MMR vaccine, a figure that is difficult to reconcile with that of the Apulian report ^3^, which found 38 serious AEFIs out of 1000 enrolled – a number almost 300 folds greater than that in the AIFA Report.

In the AIFA Vaccines Report for year 2017 ^13^, the occurrence of the different clinical reactions to the vaccine is also described ( Table 1 right column). The serious AEFI more often correlated to vaccination was hyperpyrexia, with a reporting rate of 0.108 events per 1000 doses administered. Reporting rates for other MMRV or MMR vaccination-related serious AEFIs of major interest (rate per 1000 doses) are also described: generalized skin reactions 0.02; morbilliform/varicelliform rash: 0.013; convulsions: 0.005; thrombocytopenia: 0.007; ataxia/balance disorder: 0.002. These values are clearly very different (and much lower!) from those reported in the research of the Apulia Region.

The results of the Apulian studies are also surprising with regard to serious neurological symptoms and seizures. On the one side, an incidence of serious but unspecified neurological symptoms of 20/1000 is a new and unexpected finding, on the other a rather low incidence (0.5/1000) of febrile seizures is reported. In the eight year retrospective study, not a single case of febrile seizures was documented. In previous active surveillance programs the incidence of febrile seizures following the first dose of MMRV was 2.6 per 1000 (8/3019) ^7^ or 1.7/1000 ^10^, higher than the reported risk of 0/1000 ^5^ or 0.5/1000 ^3^. In a recent Cochrane review, the attributable risk to vaccine-induced febrile seizures is estimated around 1/1700 – 1/1150 administered doses ^12^, but this value includes also MMR without varicella immunization. Stefanizzi *et al.* attribute their described low incidence both to the low reporting in the spontaneous pharmacovigilance study ^5^, and to the fact that in three of four cases they discarded the responsibility of the vaccine by applying the WHO causality assessment algorithm.

## Discussion

The data collection of the Epidemiological Observatory of the Apulia Region ^3– 5^ provided a great deal of thought-provoking results. Given the importance of the study, the only one in Italy with active surveillance combined with the WHO causality assessment, we believe it is important to discuss the authors’ interpretations of the data.

These issues are of general interest, because the different methods of detecting AEFI are under debate and because the adverse effects of MMRV vaccine are still under scrutiny ^7, 8, 12, 17, 18^. Moreover, the question of the MMRV combination vaccine versus separate administration of MMR and varicella components is open to investigation ^10^, also considering that the real need of vaccination for varicella can be discussed separately from other immunizations ^19^.

In general, the reported epidemiological data contrast with some reassuring conclusions about the safety of the vaccine and with other statements in the cited papers ^3– 5^, about the methods of pharmacovigilance of vaccines. We aim to highlight some inconsistencies in the interpretation of the observed data and to present another point of view, concluding with some proposals about resuming active vigilance programs.

## Emerging signals

The abstract of the paper of active surveillance ^3^ ends with an encouraging statement: " *Because no emerging signals were detected, our data from the active surveillance program confirmed the safety profile of the MMRV vaccine.*" Furthermore, in the Introduction, the authors state that following vaccinations " *serious AEFIs are absolutely rare*", precisely citing an Expert Opinion on Drug Safety ^20^ and the aforementioned WHO document ^15^. The Discussion reiterates that " *the active surveillance program confirmed and reinforced the safety profile of the vaccine*". The data in the article ^3^ however, are different: many readers may understand "absolutely rare" as "very rare", but internationally very rare events are defined as those with a frequency <1/10,000, while in the report the causally related serious AEFIs are 38/1000. This frequency should classify them as “common” AEFIs (<1/10 but > 1/100) ^21^.

The results of the Apulia report should be compared with what is already known by the reports of the national health authorities. The data of the active surveillance show a number of serious AEFIs related to the MMRV vaccine hundreds of times higher than expected, based on spontaneous surveillance and AIFA reports. It is surprising that 38/1000 serious AEFIs, instead of 0.127/1000 declared in the AIFA Report for the same vaccine, are not considered an "emerging signal". Although a high incidence of febrile reactions and skin rash in the first 10 days after MMRV vaccination was expected and here confirmed (but in 38 x 1000 cases they were classified as ‘serious’), serious neurological symptoms and serious gastrointestinal diseases are not described with such frequency in the literature or by current surveillance systems.

This marked discrepancy is almost certainly due to the differences between active reporting and the passive (or mixed) reporting adopted by the Italian health authorities. It is implausible that such a large discrepancy could be due to local factors, such as the use of different vaccines or different sensitivity to adverse events of the population. It cannot be excluded that an incidence of AEFIs as high as that reported ^3^ may be, in part, due to the concomitant injection of a Hepatitis A vaccine, but data to explore this possibility are not provided. Whatever the reasons, the report of the Apulia Region offers an unexpected and worrying picture of the incidence of serious AEFIs and cannot be taken as a confirmation of what already known.

Stefanizzi *et al*. ^3^ maintain that these numbers are consistent with a similar active surveillance report with telephone interviews by Huang *et al.* ^22^, in which AEFIs were 480/1000 at follow-up. However, this paper concerned an adjuvanted vaccine against H5N1 influenza and adult participants. A previous randomized controlled trial of active surveillance for the MMRV vaccine (ProQuad) ^11^ reported an AEFI rate of 65.6%, of which 37.7% were judged to be related. However, only 0.7% of the described AEFIs were classified as serious and, among them, 0.3% were judged to be related.

The data are not reassuring, and require reconsideration of the validity of the AIFA annual reports, essentially based on passive surveillance.

## Under-reporting

In the Introduction of their paper, Stefanizzi *et al.* ^3^ state that " *passive post-marketing surveillance is affected by under-reporting, especially for non-serious AEFIs*". Their Discussion asserts that “ *The proportion of non-serious adverse events resulted higher than the Italian estimate that indicated for 2017 in the last AIFA report (88.7% vs. 80.0% in the AIFA report) and higher than Apulia data from passive surveillance in the 2013–2017 period (88.7% vs. 75.4%): this findings seems to indicate that the under-report of passive AEFIs surveillance mainly regarded non-serious adverse events*” ^3^ – here the authors mention the 2018 report of the Observatory of the Apulia Region ^4^. However, the data show a different picture. While in the Apulia study ^3^ the “ *proportion*” of non-serious AEFIs detected with passive surveillance is close to the 2017 AIFA Report (albeit a little higher: 88.7% vs 80%), the absolute amount of non-serious AEFIs x 1000 doses, which in the Apulia active surveillance report are 883 in 2,149 children, is exceedingly higher than that reported in the AIFA Report for Italy in 2017 ^13^. Again, in the same report from the Observatory of the Apulia Region previously published on the institutional website (Table 3.4.3.1.) ^4^, the reporting rate of serious AEFIs with active surveillance was 40.69 per 1000 doses, with passive surveillance 0.12 per 1000 doses. The difference between active and passive surveillance was 339 times. If we consider serious AEFIs with consistent causal association with the vaccine, the ratio published on the complete Apulia report ^4 ^(paragraph 3.4.3.2.) is even more unbalanced; reporting rate 29.3/1000 doses with active surveillance, 0.03/1000 doses with passive surveillance – a 977 times difference.

It should also be taken into account that the current schedules contemplate the repetition of the MMRV vaccine at least twice in life (but perhaps more than that, as a consequence of the shorter duration of vaccination immunity compared to that resulting from the corresponding diseases, and of a lower circulation of wild viruses, with the decline of vaccine protection in the absence of natural boosts) ^19, 23^.

Given that the large discrepancies we have described in Table 1 between the AIFA reports and the active surveillance data also concern serious AEFIs, the authors' opinion that " *the under-report of passive AEFIs surveillance mainly regarded non-serious adverse events*" ^3 ^is not evidence-based.

In the Discussion it is repeated that febrile seizures are “ *the most common adverse event following the MMRV vaccine*” ^3^. However, in the data of the Apulia Region, the other causally related serious neurological symptoms were 43 times more common than seizures ( Table 1). A better definition of these serious neurological symptoms would have been appropriate.

## Incidence of clonus/febrile seizures and causality assessment

Hyperthermia following MMRV vaccination can be accompanied by febrile convulsions and this serious event is a major concern for the population. In previous active surveillance programs, the incidence of febrile seizures post dose 1 of MMRV was 2.6 per 1000 (8/3019) ^7^ or 1.7/1000 ^10^, or “high but under 2.95/1000” ^8^, values higher than the reported risk of 0/1000 ^5 ^or 0.5/1000 ^3^. In the latter paper (by Stefanizzi *et al.*), clonus/febrile seizures were detected in 4/109 serious AEFIs (reporting rate = 2/1000 follow-up), but only one episode was considered as consistently associated with vaccination, so that the reporting rate dropped to 0.5/1000. Following this kind of evidence, the authors suggested that “ *the frequency of seizure consistently associated with the MMRV vaccine is lower than those published in the previous studies*”. However, the strength of this conclusion is based on a solitary episode after the exclusion of 3/4 cases of seizures, though a causality assessment of these cases was not clearly described.

A role of vaccination in 2/4 clonus/febrile seizure episodes was excluded because of the presence of “ *alternative cause of adverse events*”. 1/4 cases was excluded as indeterminate because “ *another cause of hyperpyrexia and febrile seizure was supposed during hospitalization (viral pharyngotonsillitis)*”. In the Stefanizzi *et al.* report ^3^, the supposed “alternative causes” in 2 of 4 cases are not even mentioned, thus hindering any independent evaluation of the circumstance.

We should also consider that causality assessment was carried out by two public health physicians, experts in vaccinology, not by neurologists or pediatricians or pathologists, while to ensure compliance with the rigorous criteria for causality assessment and wider acceptance of the results, the WHO manual ^15^ recommends that the procedure is performed by a multidisciplinary committee comprising experts from pediatrics, neurology, general medicine, forensic medicine, pathology, microbiology, immunology and epidemiology.

The text deals with “clonus/febrile seizures”, without clearly distinguishing between the two conditions. However, it is known that the most frequent convulsions not related to hyperthermia are of the tonic-clonic type and not only clonic. Moreover, assuming that the "alternative causes" of the seizures were epilepsy, it would be important to consider the relationship between epilepsy and specific vaccines, since vaccination might precipitate adverse events in children with familial tendency to seizures or genetic epilepsy syndrome ^2, 24– 26^. It would not be conceptually and scientifically correct to use the WHO algorithm in such a way as to consider an "alternative cause" a genetic tendency to epilepsy, because the vaccine in this case could be the precipitating or triggering factor of the individual's susceptibility. Recognition of this possible occurrence is important, because there is a need to select vaccines that carry lower risk of febrile seizures in these children who are particularly prone to develop this adverse event ^27^.

Especially for infectious and inflammatory illnesses, it has been noted that the supposed “other cause”, mentioned by WHO algorithm, should be *independent* from a possible interaction with the perturbation induced by vaccination ^1, 2^. In fact, vaccination can act as a synergistic or triggering factor in a person affected by genetic susceptibility or by a concomitant infectious disease. None of these issues is addressed in the cited paper ^3^. Concerning the case of seizures in a child with “viral pharyngotonsillitis”, this diagnosis does not exclude the contribution of vaccination in the development of hyperpyrexia and seizures. A concomitant infectious or inflammatory disease occurring in the time window of the immune reaction to vaccine cannot make the role of vaccine “indeterminate” and so a causality link cannot be excluded ^2^.

Because of the extremely small sample of the study and of the outlined doubts about the exclusion of the three cases, it seems highly questionable the conclusion ^3^ that the frequency of seizure consistently associated with the MMRV vaccine “ *is lower than those published in the previous studies that considered all seizure temporary associated with the vaccination, without a standardized causal evaluation*”.

To illustrate how the application of the WHO algorithm is difficult and potentially error-prone, four case studies are presented ( Box 1), in which the causality association of a serious neurological AEFI with the vaccine was excluded ^5^. These examples raise some problems and deserve clarification, without which a high risk of misinterpretation exists. The notes of the authors concern: a) whether the alternative "other cause" was sufficiently clear and "strong" as the only possible cause of the event and b) whether there could be a plausible interaction between other clinical conditions and the biological action of the vaccine.

**Box 1. Serious neurological adverse events following measles-mumps-rubella-varicella vaccine reported by the Apulia studies ^3, 5^ and comments on causality assessment**

**Case 1**

Case n. 9 cited in the paper of Stefanizzi *et al.* ^5^: *"The ninth case involved a 12-months-old female. A week after the vaccination, she presented a sudden loss of consciousness with staring eyes, hypertonic for about 10 min, modest hypersalivation. She was hospitalized and, after medical examination, she was discharged with the diagnosis of hyporesponsive episode in patient with vomiting and metabolic acidosis. Applying the Causality Assessment algorithm, cause/effect relationship between vaccination and adverse events is inconsistent, because an alternative cause (gastrointestinal infectious disease) has been recognized."*

Note: In this case, the adverse effects following immunization (AEFI) took place precisely in the time window in which the greatest number of episodes of febrile seizures normally occur, so there is a high biological plausibility and a correct time window for attributing causality to the vaccine. In the report by Stefanizzi *et al.* ^3^ there is a very high incidence of serious gastrointestinal symptoms with a causality ascertained with the vaccine. It is not possible to understand how vomiting and metabolic acidosis can justify the diagnosis of "gastrointestinal infectious disease" as an “alternative cause”, also without a microbiological analysis. Notably, according to the first step of World Health Organization (WHO) algorithm of causality assessment ^15^, when the AEFI occurs in the expected time window and there is biological plausibility, a supposed “other cause” must be “strong” enough to exclude the role of the vaccine in the causality. This criterion does not appear to support the attribution of neurological symptoms to a supposed “gastrointestinal infectious disease” rather than to a vaccine adverse reaction. Furthermore, even if it were really a gastroenteritis, it cannot be excluded that the neurological symptoms were due to the perturbation of the gut-brain axis ^28^, that is, in our case, to the interaction between the induced inflammatory stress from the vaccine and gastrointestinal disorder with alteration of the mucosa, release of endotoxins or other metabolites in the circulation ^2^.

**Case 2**

Case cited in both reports ^3, 5^: " *The 13th case regarded a 15-months-old male. Nine days after vaccination, he reported hyperpyrexia and febrile seizure associated with eyes rolling, limbs twitchings, and loss of consciousness. This episode ended after a few minutes: for these symptoms, he was admitted to the hospital and discharged after 3 days for the complete AEFI resolution. During hospitalization he presented fever but he did not report another episode of febrile seizures. After medical examination, a final diagnosis of febrile seizure caused by viral pharyngotonsillitis was formulated. Applying the Causality assessment algorithm, the cause/effect relationship between vaccination and adverse events is inconsistent for the presence of an alternative disease (viral pharyngotonsillitis).*”

Note: In this case the febrile convulsions occurred in the most probable time window and there is also a considerable literature on the fact that the vaccine can cause this phenomenon. The concomitant presence of pharyngotonsillitis cannot be considered an alternative cause strong enough to rule out the role played by the hyperpyrexia caused by the vaccine. In this case, a trivial viral infection could well have occurred in a child whose immune system was very stressed by vaccination with four live viruses and the strong fever due to the two different causes may have triggered the seizures. It is notoriously recommended not to vaccinate a person if he has another infectious disease in progress, but if the vaccination takes place during the period of incubation of the infection, a pathological synergy between the two stimuli may occur. Another possibility that cannot be ruled out, at least in principle, is that the pharyngotonsillitis was caused directly by one of the injected live vaccine viruses. It is known that the measles vaccine virus infects lymphatic tissue ^29^ and vaccine-related upper respiratory infections are reported in 12/1000 of children vaccinated with MMRV (ProQuad) ^30^. Incidentally, the causal assessment decision for the same case (viral pharyngotonsillitis and post-MMRV seizures) was judged as "indeterminate" in one case ^3^ and "inconsistent" in a subsequent publication ^5^, but the two classifications are very different according to the same WHO manual.

**Case 3**

Case n. 19 cited in the paper of Stefanizzi *et al.* ^5^: “ *The case involved a 15-months-old female vaccinated with MMRV and anti-HAV vaccines. Ten days after immunization, she developed fever and hyperpyrexia and strabismus, which was classified as serious and permanent invalidity. Applying the Causality Assessment algorithm, the cause/effect relationship between vaccination and adverse events is not consistent, because of the absence of biological plausibility between strabismus and vaccine administration*.”

Note: in this case, it does not seem correct to exclude a causal relationship with vaccination by appealing to the lack of biological plausibility. In fact, strabismus may be caused by oculomotor nerve palsy ^31^ and several cases of third cranial nerve palsy after vaccination (with both live and inactivated vaccines) have been described and reported in the US Vaccine Adverse Event Reporting System (VAERS) ^32^. Although it is not possible to determine causal associations based on VAERS reports, the authors of the review do not deny it either. More importantly, they do not question the plausibility of such an adverse reaction, because cranial nerve palsy may sometimes be the harbinger of encephalomyelitis, which may, although rarely, be caused by vaccinations. Cases of oculomotor nerve palsy occurring after MMR vaccination has already been described in the scientific literature ^33, 34^.

**Case 4**

Case n. 23 cited in the paper of Stefanizzi *et al.* ^5^: “ *The case involves a male child aged 30 months: few hours after vaccination, he developed hyperpyrexia with an episode of febrile seizure. He was hospitalized and symptoms persisted for 9 days. Applying the Causality Assessment algorithm, the cause/effect relationship between vaccination and adverse events is classifiable as inconsistent: even the biological plausibility of AEFI, the time window between vaccination and adverse reactions (hyperpyrexia and febrile seizure) is not compatible (too short*).”

Note: Although hyperpyrexia caused by MMRV vaccine usually peaks after one week from the first dose in about 10% of subjects, in some subjects it occurs between the first and the 5th day after inoculation. In a randomized study with active surveillance ^11^ it was observed that the rate of fever (temperature > 39.0° C) in the first 5 days after first dose of MMRV was 8 cases every 1000 doses. This data makes it improper to exclude causation in a case of febrile seizures by applying only a weak criterion such as a time window that excludes the first day after the vaccine injection.

## MMRV or MMR+V?

A related point, not discussed in the cited paper, concerns the formulation of the vaccine used. An increased risk of onset of fever and seizures has been documented after administration of the first dose of tetravalent vaccine (MMRV) compared to separate administration of MMR vaccines and varicella vaccine ^35– 39^. Analysis on the Italian national database of AIFA confirmed a more than two-fold risk of febrile seizures after administration of MPRV vaccine compared to vaccination with separate vaccines. This finding particularly applies to younger children and it is mostly observed in the first 5-12 days after the administration of the vaccine. Therefore, the AIFA Paediatric Working Group, similarly to what is suggested in other countries, recommended pediatricians and other health professionals not to use the tetravalent MPRV vaccine as a first dose for immunization against measles, mumps and rubella ^40^. Furthermore, most of the children who participated in the active surveillance research ^3^ were inoculated with MMRV vaccine plus hepatitis A vaccine, but a potential extra effect of simultaneous vaccination in the same session cannot be inferred from the aggregated results. These important issues, not considered in the study, make even more doubtful the interpretation of the relatively low rate of febrile seizures in the sample from Apulia.

In this context, it seems useful to extend our attention to the choice among different vaccines and even to the choice of whether to vaccinate or not, a choice that must be made by the patient or by parents of underage children, properly advised by their doctors. Informed consent and/or the refusal of therapy by patients (or by parents in case of pediatric vaccinations) are debated issues in the medical profession and their importance is underlined by various international statements, such as the Convention on Human Rights and Biomedicine (ETS No 164) signed the 4 April 1997 in Oviedo (Spain). The introduction in the legislation of many countries of an obligation to be vaccinated has significantly altered the patient-doctor and parents-doctor relationships. Regardless of the different vaccines being useful or not, the rare possible single exemptions provided by the law, if there is a legal obligation the doctor’s role risks to switch from an "evidence-based" counsellor to a public officer enforcing the government’s decisions. Such issues were further exacerbated by the introduction of multiple component vaccines, such as the tetravalent with live attenuated viruses or the hexavalent with antigens fixed on aluminum nanoparticles. In these cases the doctor was also denied the possibility of recommending the most appropriate choice based on the epidemiological conditions and on the characteristics of the individual patient's susceptibility. The Italian law issued in July 2017 (law 119/2017) mandated that the vaccination obligation for MMRV should be revised after three years, but so far nothing has been done and nothing is expected to be done in the near future. Although these issues are still open, it is agreed that patients consulting their doctors for vaccination advice should be offered correct information of the highest qualitative and quantitative standard. This is why the principle of informed consent does not seem satisfied by a generic statement of a "safe profile" or "lack of worrying signals" of a vaccine, that indeed has shown serious adverse effects in a significant percentage of subjects. We hope our contribution may be useful to help the medical professionals and especially the pediatricians to evaluate the information to be shared with the public by utilizing authentic scientific evidence.

## Perspectives

The Apulia epidemiological studies ^3– 5 ^have the merit of clearly raising the issue of methods for detecting adverse reactions, a crucial aspect of vaccinology. We agree with the conclusion of the article ^3^ which states that " *active surveillance programs periodically have to be implemented in order to improve the overall performance of the pharmacovigilance system and validate the data and emerging signals detected by spontaneous reporting activity*"; and that " *active surveillance programs can be considered an effective solution to a real question: the public concerns about risks associated with immunization*." However, in explaining this legitimate objective, the authors do not notice that the reiterated reassurance about safety problems, in line with the dominant paradigm, is not consistent with the results of their active surveillance. These results are objectively not so reassuring due to the serious AEFIs carefully documented at the individual level and their possible impact projected at the level of the national community.

The debate on the best methods of surveillance in the field of vaccinology should remain open, in the interest of the entire population ^1, 2, 41– 43^. Besides the WHO algorithm ^15 ^other criteria developed by various groups working within the greater field of pharmacovigilance are used for causality assessment, such as the World Health Organization Collaborating Centre for International Drug Monitoring, the Uppsala Monitoring Centre (WHO–UMC) ^44^ and the Naranjo algorithm ^45, 46^. Unlike the WHO algorithm, which judges as inconsistent the association of an AEFI with vaccination if there is “another cause”, the other methods use a score or a probability assessment, taking into account the clinical-pharmacological aspects of the case history and the quality of the clinical documentation. This approach seems more suitable for judging complex cases, in which multiple interacting causes (genetic, infectious, toxic) determine the pathology that occurs after vaccination. Moreover, the same level of evidence for assessing causality should be applied to the alternatively proposed causal factors. The mere mention of a possible alternative causal factor is not evidence strong enough for ruling out vaccines.

The cited research highlights the inadequacy of passive surveillance to represent the real incidence of even short-term AEFIs, both of mild and serious kind. The distance between the incidences of AEFIs detected ^3^ and the passive reports collected in the same Region ^5^ and even in the Italian regions with the most efficient reporting system ^13, 14^ calls into question the extent of investments to improve spontaneous reports. These reports must be maintained, to allow the reporting of rare events that active surveillance would have little chance to intercept. Moreover, investments should be redirected to active surveillance studies (indeed already committed by the Italian Ministry of Health), designed on representative samples of the Italian population, the only ones from which valid and credible inferences can be drawn. In any event, the public reports should present the active and passive surveillance data in a disaggregated way.

The publication of the Apulia active surveillance experience has also the merit of having applied the WHO algorithm for causality assessment ^15^, but this important step of analysis was applied in a debatable way. In light of these important questions, we believe it is appropriate to open a scientific debate, also taking into account critical and constructive scientific positions, based on the available set of evidence about effectiveness and safety of current immunization schedules, on a continuous reassessment of their validity, and on the frank admission of the persistent areas of uncertainty. We deem also legitimate an open scientific debate about possible different implementation strategies and priority assessments among the current immunization strategies.

A contribution to evaluate the benefits and risks of vaccines, including long-term ones, could come from comparative studies between vaccinated and unvaccinated groups of children. This type of study cannot be realized in randomized groups for ethical reasons, but preliminary data can be collected anyway from accurate observational studies comparing normally vaccinated children with those who refused one or more vaccines. Unfortunately, few studies of this type are known. Here we mention those of Aaby's group ^47– 49^, who reported very different results according to the types of vaccines and regions of the world where the studies were conducted, and those of Hooker's ^50^ and Lyons-Weiler ^51^. In the first study ^50^ the children were vaccinated before 1 year of age and then evaluated when they reached a minimum age of 3 years. The vaccination was associated with significant higher odds of developmental delays (OR = 2.18), asthma (OR = 4.49) and ear infections (OR = 2.13). However, the study only allowed the computation of unadjusted observational associations. Higher odds ratios for such diseases were observed in quartiles where more vaccine doses were received than in quartile 1. In the second study ^51^ it was performed a multicenter retrospective analysis covering ten years of pediatric practice and the incidence of office visits, noting the various pathologies motivating them. The ORs indicated a notable increase in outpatient visits in vaccinated compared with unvaccinated children, because of anemia (OR = 6.3), asthma (OR = 3.5), allergic rhinitis (OR = 6.5) and sinusitis (OR = 3.5). Despite the limitations of this type of investigation and the impossibility of causal inferences, the overall results suggest that vaccinated pediatric patients are no healthier than unvaccinated ones. Further studies are needed to follow vaccinated and non-vaccinated cohorts prospectively, to understand the full spectrum of health effects associated with childhood vaccination.

Finally, we want to relaunch a proposal ^52^ to investigate the possible “non-specific effects” of vaccines. The current pharmacovigilance systems are not apt to identify such effects, the observational studies cannot establish causality, and the few randomized clinical trials (RCTs) do not have sufficient size and follow up to identify and prove causality of rare or uncommon non-specific effects, even more so if they have a sizeable background rate. A solution might take advantage on the vaccine hesitancy, that remains in some individuals even after receiving an extensive and balanced information, based on the state of knowledge ^52^. These persistently hesitant individuals (for themselves or for their children) could be tens of thousands in a country: they could be considered as a valuable asset to society, offering them the opportunity to participate in properly designed and long-lasting RCTs. The current ethical barriers to such RCTs (the exclusion of the randomized control groups from the vaccine benefits) could be overcome because these subjects are typically evenly dispersed in a country (posing negligible risk for immunocompromised individuals ^23^), are really and persistently hesitant and may be willing to give their informed consent to participate in such RCTs. So this experimental approach could contribute to an advance in the scientific

## Data availability

All data underlying the results are available as part of the article and no additional source data are required.
