# Supplementary material for: Adverse events following measles-mumps-rubella-varicella vaccine: an independent perspective on Italian pharmacovigilance data
Source: F1000Res. 2021 Jan 6;9:1176. Originally published 2020 Sep 28. [Version 2] doi: 10.12688/f1000research.26523.2 (PMC7721067; doi:10.12688/f1000research.26523.2)
Supplement: Peer review history

# Peer review: Reviewer response for version 1

**DOI:** 10.5256/f1000research.29282.r75611

**Published:** 2020-12-03

**Authors:** James Lyons-Weiler

**Affiliations:** 1 The Institute for Pure and Applied Knowledge, Pittsburgh, PA, USA

This article finds evidence of adverse events associated with the MMRV Vaccine in a post-market surveillance study.

 The authors have applied the WHO criteria for causality and critique a previous report for underreporting, subjective exclusion of Day 1 events, and provides four examples of arbitrary or subjective exclusion of specific cases that under more objective criteria meet the criteria of evidence in support of causality.

 The authors have found increased incidence of severe hyperpyrexia, neurological and gastrointestinal diseases compared to the prior report, but provide no reference to national or regional baseline rates of these conditions in age-matched children that could not have received the MMRV vaccine. Such data may not exist, and that would be failing of public health data reporting, not the authors. However, if such data do exist, it would serve well to include references to rates of such conditions in age-match (or close-to-age matched) unvaccinated children.

 There are some new studies that are worth citing that might be considered. To avoid any COI I do not list my own. I recommend for example Hooker and Miller ^1^ and Peter Aaby's group's recent studies (especially the DTaP study in a vaccine-naïve population) ^2^.

 The authors are correct in their views that "This approach seems more suitable for judging complex cases, in which multiple interacting causes (genetic, infectious, toxic) determine the pathology that occurs after vaccination."

 However, they should also consider that the same level of evidence for assessing causality should be applied to alternatively proposed causal factors. The mere mention of a possible alternative causal factor is not strong evidence ruling out vaccines.

 Further, they might consider outlining the potential reality that causal interactions exist between vaccines and the proposed alternative factors as risk factors for vaccine adverse event should also not be ignored.

 Overall the writing is truly excellent, there are few sections that repeat similar information, they authors might consider combining a couple of paragraphs separated by others.

 I strongly recommend this report being indexed, with minor updates from the author.

Are arguments sufficiently supported by evidence from the published literature or by new data and results?

Yes

Is the conclusion balanced and justified on the basis of the presented arguments?

Yes

Is the rationale for commenting on the previous publication clearly described?

Yes

Are any opinions stated well-argued, clear and cogent?

Yes

Reviewer Expertise:

I have conducted research on biomarkers of adverse events from medical treatments in cancer, published studies that have determined pediatric dose limits and excess of aluminum in vaccines in the CDC's schedule, catch-up schedule, and an alternative schedule, and have recently conducted a vaccinated vs. unvaccinated study in a pediatric population looking at numerous health outcomes. My training is in biology, computational molecular biology, and my professional work experience has been academic research in biomedicine focused on differential diagnosis and data-aided prognosis of health outcomes since 2000.

I confirm that I have read this submission and believe that I have an appropriate level of expertise to confirm that it is of an acceptable scientific standard.

## References

[1] : Analysis of health outcomes in vaccinated and unvaccinated children: Developmental delays, asthma, ear infections and gastrointestinal disorders. *SAGE Open Med*.2020;8: 10.1177/2050312120925344 2050312120925344 10.1177/2050312120925344 32537156PMC7268563

[2] : Evidence of Increase in Mortality After the Introduction of Diphtheria-Tetanus-Pertussis Vaccine to Children Aged 6-35 Months in Guinea-Bissau: A Time for Reflection?. *Front Public Health*.2018;6: 10.3389/fpubh.2018.00079 79 10.3389/fpubh.2018.00079 29616207PMC5868131

# Response

**Published:** 2020-12-22

**Authors:** Paolo Bellavite

**Affiliations:** University of Verona School of Medicine, Italy

We thank the reviewer for these positive notes and suggestions, covering two aspects that we have implemented in the secnd version. The reviewer points out that in order to correctly assess the relationship between benefits and risks of a vaccination it would be important to be able to compare the baseline health status of vaccinated versus unvaccinated children. Unfortunately, these data are lacking because systematic and adequate studies are not carried out on pediatric populations. These studies which would be feasible without too much difficulty if we had require efficient registry functions, capable of documenting the vaccination and health status informing about vaccinations and the state of health of the population. However, there are some observational studies that certainly deserve to be cited, because they can give general indications, even with in the presence of inevitable biases related to the study design. Therefore, we have briefly cited some research, such as that of Hooker-Miller and of the Aaby's group. We also mentioned Weiler's study, although he did not correctly ask for it, because it is a very relevant contribution in this context: “A contribution to evaluate the benefits and risks of vaccines, including long-term ones, could come from comparative studies between vaccinated and unvaccinated groups of children. This type of study cannot be realized in randomized groups for ethical reasons, but preliminary data can be collected anyway from accurate observational studies comparing normally vaccinated children with those who refused one or more vaccines. Unfortunately, few studies of this type are known. Here we mention those of Aaby's group ^47-49^, who reported very different results according to the types of vaccines and regions of the world where the studies were conducted, and those of Hooker's ^50^ and Lyons-Weiler ^51^. In the first study ^50^ the children were vaccinated before 1 year of age and then evaluated when they reached a minimum age of 3 years. The vaccination was associated with significant higher odds of developmental delays (OR = 2.18), asthma (OR = 4.49) and ear infections (OR = 2.13). However, the study only allowed the computation of unadjusted observational associations. Higher odds ratios for such diseases were observed in quartiles where more vaccine doses were received than in quartile 1. In the second study ^51^ it was performed a multicenter retrospective analysis covering ten years of pediatric practice and the incidence of office visits, noting the various pathologies motivating them. The ORs indicated a notable increase in outpatient visits in vaccinated compared with unvaccinated children, because of anemia (OR = 6.3), asthma (OR = 3.5), allergic rhinitis (OR = 6.5) and sinusitis (OR = 3.5). Despite the limitations of this type of investigation and the impossibility of causal inferences, the overall results suggest that vaccinated pediatric patients are no healthier than unvaccinated ones. Further studies are needed to follow vaccinated and non-vaccinated cohorts prospectively, to understand the full spectrum of health effects associated with childhood vaccination.”

 As a new contribution to this important issue, we have added a final paragraph with a suggestion for possible comparative studies between vaccinated and unvaccinated, taking advantage of the known and frequent phenomenon of "vaccine hesitancy": “Finally, we want to relaunch a proposal ^52^ to investigate the possible “non-specific effects” of vaccines. The current pharmacovigilance systems are not apt to identify such effects, the observational studies cannot establish causality, and the few randomized clinical trials (RCTs) do not have sufficient size and follow up to identify and prove causality of rare or uncommon non-specific effects, even more so if they have a sizeable background rate. A solution might take advantage on the vaccine hesitancy, that remains in some individuals even after receiving an extensive and balanced information, based on the state of knowledge ^52^. These persistently hesitant individuals (for themselves or for their children) could be tens of thousands in a country: they could be considered as a valuable asset to society, offering them the opportunity to participate in properly designed and long-lasting RCTs. The current ethical barriers to such RCTs (the exclusion of the randomized control groups from the vaccine benefits) could be overcome because these subjects are typically evenly dispersed in a country (posing negligible risk for immunocompromised individuals ^23^), are really and persistently hesitant and may be willing to give their informed consent to participate in such RCTs. So this experimental approach could contribute to an advance in the scientific.”

 Finally, the reviewer also noted that our sentence "This approach seems more suitable for judging complex cases, in which multiple interacting causes (genetic, infectious, toxic) determine the pathology that occurs after vaccination." it is itself correct, but incomplete. We agree with this opinion and therefore we have modified it by expressing better the concept, as he suggested, in the "Perspectives" section: “This approach seems more suitable for judging complex cases, in which multiple interacting causes (genetic, infectious, toxic) determine the pathology that occurs after vaccination. Moreover, the same level of evidence for assessing causality should be applied to the alternatively proposed causal factors. The mere mention of a possible alternative causal factor is not evidence strong enough for ruling out vaccines.”

# Peer review: Reviewer response for version 1

**DOI:** 10.5256/f1000research.29282.r75394

**Published:** 2020-11-26

**Authors:** Arthur Brawer

**Affiliations:** 1 School of Medicine, Drexel University, Philadelphia, USA; 2 Robert Wood Johnson Medical School, New Brunswick, USA

Vaccination-induced disorders are a genuine reality that continue to generate intense controversy. Although the majority of immunization recipients have little or no safety issues, that does not detract from the occurrences of multiple chronic systemic diseases initiated by a wide variety of parenteral vaccine exposures. Over the past four decades case reports of chronic vaccination-induced disorders published in peer-reviewed journals have generally segregated into two main categories: (a) autoimmune and autoinflammatory diseases; and (b) neuro-psychiatric diseases, with or without seizures and/or dyskinesias, and with or without overlapping clinical features resembling the various neurologic fatiguing syndromes (e.g. chronic fatigue syndrome, dysautonomia, small fiber neuropathy, fibromyalgia, and postural orthostatic tachycardia syndrome). Recently some novel ideas have been proposed regarding definitive identification of those at risk for any of the above phenomena in either category.

 Passive surveillance programs to monitor vaccine safety and effectiveness rely on reporting by patients, family members, manufacturers, and health care providers to capture either a serious or minor adverse event that has temporally occurred after immunization. There are many inherent problems with such programs, not the least of which are: (1) you can’t analyze what hasn’t been reported; (2) you can’t analyze what has been frivolously labelled as unrelated; and (3) you can’t analyze something serious that has been labelled trivial. Active surveillance programs utilize health care encounters and electronic medical records to capture data and are generally better in detecting safety signals in real time. However, as pointed out by the authors, such statistical signals can be spurious for many reasons. In addition, mechanisms of disease causation that initiated an acute severe vaccination-related event can subsequently be accompanied weeks later by other latent mechanisms that evolved more slowly before becoming clinically relevant. These secondary amplification loops can circuitously augment and perpetuate an acute severe adverse event and turn it into a chronic disabling process. Months later another physician may erroneously interpret the two processes to be separate events, whereby valid detection and assignment of the initial adverse immunization reaction are nullified. A specific example of this process is the initiation of a serious adverse event by hidden toxic chemicals that routinely accompany the beneficial ingredients in dozens of different parenteral vaccines. Such chemicals are also capable of inducing the latent development of multiple autoantibodies, the latter of which then transform the initial acute event into a chronic disease. 

 Children whose parents refuse or delay vaccinations are often dismissed from ongoing care by pediatricians. Likewise, vaccine refusal by adult patients can trigger the same fate from their internist. Authors Bellavite and Donzelli present valid arguments favoring reassessment of the safety data and effectiveness of current immunization schedules. These reassessments can serve as a template for clinicians of all specialties to reexamine this issue and not merely dismiss parental worries as unfounded hysteria. I favor this manuscript being indexed, as it will also likely induce academicians to reassess their denials of vaccination-induced diseases and refocus their attention towards defining the population at risk for serious adverse events.

Are arguments sufficiently supported by evidence from the published literature or by new data and results?

Yes

Is the conclusion balanced and justified on the basis of the presented arguments?

Yes

Is the rationale for commenting on the previous publication clearly described?

Yes

Are any opinions stated well-argued, clear and cogent?

Yes

Reviewer Expertise:

I am a board certified rheumatologist who has published many peer-reviewed manuscripts encompassing vaccination-induced disorders.  I am qualified to testify in vaccine court in Washington, D.C., where special masters adjudicate claims of vaccine related ailments.

I confirm that I have read this submission and believe that I have an appropriate level of expertise to confirm that it is of an acceptable scientific standard.

# Response

**Published:** 2020-12-22

**Authors:** Paolo Bellavite

**Affiliations:** University of Verona School of Medicine, Italy

We thank the Reviewer for these notes, confirming and deepening some points of our work. In particular, we agree on the emphasis on informed consent, sometimes underestimated by pediatricians, especially because of the laws that oblige parents to have fully vaccinated children undergo all vccinations, under penalty of exclusion from nursery school or sanctions. Therefore, we have included in the discussion a paragraph about this issue, as follows: “In this context, it seems useful to extend our attention to the choice among different vaccines and even to the choice of whether to vaccinate or not, a choice that must be made by the patient or by parents of underage children, properly advised by their doctors. Informed consent and/or the refusal of therapy by patients (or by parents in case of pediatric vaccinations) are debated issues in the medical profession and their importance is underlined by various international statements, such as the Convention on Human Rights and Biomedicine (ETS No 164) signed the 4 April 1997 in Oviedo (Spain). The introduction in the legislation of many countries of an obligation to be vaccinated has significantly altered the patient-doctor and parents-doctor relationships. Regardless of the different vaccines being useful or not, the rare possible single exemptions provided by the law, if there is a legal obligation the doctor’s role risks to switch from an "evidence-based" counsellor to a public officer enforcing the government’s decisions.  Such issues were further exacerbated by the introduction of multiple component vaccines, such as the tetravalent with live attenuated viruses or the hexavalent with antigens fixed on aluminum nanoparticles. In these cases the doctor was also denied the possibility of recommending the most appropriate choice based on the epidemiological conditions and on the characteristics of the individual patient's susceptibility. The Italian law issued in July 2017 (law 119/2017) mandated that the vaccination obligation for MMRV should be revised after three years, but so far nothing has been done and nothing is expected to be done in the near future. Although these issues are still open, it is agreed that patients consulting their doctors for vaccination advice should be offered correct information of the highest qualitative and quantitative standard. This is why the principle of informed consent does not seem satisfied by a generic statement of a "safe profile" or "lack of worrying signals" of a vaccine, that indeed  has shown serious adverse effects in a significant percentage of subjects. We hope our contribution may be useful to help the medical professionals and especially the pediatricians to evaluate the information to be shared with the public by utilizing authentic scientific evidence.”
